# Gastronomic Paradigms in Contemporary Western Cuisine: From French Haute Cuisine to Mass Media Gastronomy

**DOI:** 10.3389/fnut.2019.00192

**Published:** 2020-01-10

**Authors:** Raimundo G. del Moral

**Affiliations:** ^1^Department of Anatomical Pathology, Hospital San Cecilio, University of Granada, Granada, Spain; ^2^Instituto de Investigación Biosanitaria de Granada, Granada, Spain

**Keywords:** gastronomic and culinary materials, cuisine, paradigm analysis, food texture, history of food culture

## Abstract

Thomas Kuhn brings the concept of “paradigm” as the fundamental engine in the progress of humanity. Kuhn defines paradigms as the set of formal theories, experiments and work methods that define a process (scientific, economic, social, and in this case, contemporary haute cuisine), in a given time. According to Kuhn, progress does not happen by gradual accumulation of knowledge, but rather by abrupt advances. In this review, the conceptual evolution experienced by contemporary gastronomy (from the French Revolution to today) is analyzed applying the paradigm structure in light of the scientific knowledge of each era. It is thus reviewed how the first and second gastronomic paradigms, proposed, respectively, by Carême and Escoffier and based on food smell and taste the first and the flavor of the sauces the second, have been replaced by the third and fourth paradigms, led, respectively, by the Nouvelle Cuisine in France and Ferrán Adriá in Spain, and in whose development touch, sight, and sound have become increasingly prominent in gastronomic pleasure. Finally, it is analyzed how new trends in gastronomy: (1) worldwide diffusion of the Spanish tapas; (2) globalization of ethnic and fusion cuisines; (3) the growing disappearance of the professional gastronomic critique and its replacement by mass media influencers; (4) the increase of sustainability in cooking with development of local-, vegan-, and paleo-cooking, comfort, and smart food and finally, (5) the growing role of social networks and the self-itis linked to photography or foodstagramming in the gastronomic experience are leading toward a new gastronomic paradigm based on the global socialization of classic gastronomy.

## Introduction

In his book The Structure of Scientific Revolutions, Thomas Kuhn brings the concept of “paradigm” as the fundamental engine of humanity's progress: the set of formal theories, experiments, and work methods that define a process (scientific, economic, social or, in this case, contemporary haute cuisine), at a certain time (1). In general, the sciences are, at any given time, governed by a single paradigm, and according to Kuhn the progress of knowledge does not happen gradually by the accumulation of contributions by scientists but, rather, the advances are made abruptly from the emergence of great scientists who suggest new theories and research techniques that break radically with the old paradigm and replace it with a new one.

Between a scientific laboratory and haute cuisine there are remarkable technological and procedural similarities (2). In the former, in addition to theories, there are researchers, reagents and specific analytical methods to demonstrate hypotheses; in the latter, there are established culinary doctrines, chefs, ingredients, and recipes that, to the satisfaction of the diner, are applied in accordance with the current paradigm, without forgetting that the kitchen has also witnessed the increasingly evident development of sophisticated methods and instruments of work (3).

Like in normal science (4) a “normal chef,” who follows the current culinary paradigm, should not criticize it, because they are trained to be effective in the kitchen, and thus unaware of the inconsistencies that the paradigm poses. When serious anomalies or new pressing social needs arise, the active paradigm is weakened and enters into “crisis.” Then, from the mind of some great cook emerges a new paradigm as a revolutionary solution for the given problem. Then, the gastronomic community ends up adhering to the new paradigm, supporting a renewed “normal cuisine.”

The role of the different senses to appreciate the organoleptic (acting on, or involving the use of, the sense organs) qualities of foods has changed markedly with the progress of civilization. Until the 1960s, gastronomic pleasure was mainly linked to the flavor of foods (see below) through the participation of chemo- and somatosthetic receptors, including those specific for glutamate and amino acids or *umami*. From the 1960s onward, first the incorporation of sight, touch, and sound to the gastronomic pleasure, and then the progressive understanding of the significant complexity associated to a meal (5) have markedly influenced the culinary pleasure of twenty first century gastronomy (6–8).

Although Kuhn's paradigm theory has already been referred to as a good tool for analyzing the evolution of contemporary cuisine (9), up to now it has not been used in a purposive way to examine the technological and ideological evolution that has occurred in cuisine and its relationship with the sensory perception of food. The object of this review is to analyze the conceptual and sensorial evolution of contemporary gastronomy (from the French Revolution to today) applying Kuhn's paradigm structure. A brief summary is shown in Tables 1, 2.

**Table 1:** Main gastronomic contributions of the first and second gastronomic paradigms.

**SECTION A: MAIN CONTRIBUTIONS OF ANTONIN CARÉME (2, 5, 6, 13, 14):**
Revision and systematic testing of multiple ingredient combinations, permutations, and mixtures to create multitude of new recipes
The pot a feu (meat stew with vegetables) and related broths are transferred to the academic haute cuisine
Creation of the first experimental kitchen laboratory in history
Preparation of the first Cooking Manuals for the people
Creation of the training, methodological, and documentary foundations of the professional cook
**SECTION B: MAIN CONTRIBUTIONS OF AUGUSTE ESCOFFIER (3, 6):**
Simplification of kitchen work with the creation of the *brigades* and *parties de cuisine*
Realization of the first restaurant menu
Reduction of the number of menu dishes to an average of 4
Substitution of the French service (all the dishes served simultaneously on the table) for the Russian service (dishes are served consecutively, following a preset order in the menu)
Preferential inclusion of seasonal foods in the menu
Finalization of dishes at the table, such as the still current steak tartare
The organization of the table service around the sauce boat with the waiter carving, adding sauce and garnishing foods with the ultimate purpose of highlighting the aromas and flavors of each recipe

**Table 2:** Main gastronomic contributions of the third and fourth gastronomic paradigms.

**SECTION A: MAIN CONTRIBUTIONS OF THE FRENCH NOUVELLE CUISINE (6):**
Drastic reduction in the number of dishes available on the menu
Substitution of Escoffier's terminology for dishes' names that are impregnated with feeling, evocation, and poetry, based on their ingredients
Use of innovative fish and seafood cuts (i.e., carpaccio), instead of traditional transversal slices
Dishes are composed of contrary ingredients, such as land and sea (meat and fish, foie gras, and vegetable salad)
Sauces are lightened and flour, butter, cream, and eggs are eliminated to favor its digestion
Introduction of ingredients not used before in haute cuisine, such as wild aromatic herbs, new spices, tropical fruits, and very low-priced fish and vegetables
Incorporation into haute cuisine of traditional Mediterranean and oriental recipes (risotto, tajin, sashimi, etc.)
The chef becomes the restaurant's lead figure to the detriment of the maître and waiters, whose protagonism is diminished
Recipes are no longer completed at the table, and the dishes are presented fully finished (Japanese style service), and even covered by a bell, seeking to surprise the diner
Increase in the number of menu dishes, introducing an extensive sampling of minimal portions for each dish, seeking to limit caloric intake, thus making the long and narrow menu compatible with a sedentary lifestyle (*cuisine minceur*)
End of thick sauces, with a reduction of ingredient cooking time, even introducing raw foods, in search of maximum naturalness and preservation of their nutritional qualities
**SECTION B: MAIN CONTRIBUTIONS OF ABSTRACT GASTRONOMY (18–21, 25, 31–33):**
Food deconstruction aims to change its texture without diminishing its aroma and flavor
The creation of new food textures becomes one of the sensory keys of gastronomic pleasure
The introduction in haute cuisine of new texturizing and emulsifying molecules of industrial origin as ingredients
Involvement of the senses of sight (edible landscaping) and hearing in gastronomic pleasure
Food reconstruction pursues a radical modification of its flavor (melon caviar) or its texture (spherical olives, agar-agar spaghetti)
The introduction of liquid nitrogen to create new textures and temperatures
Development of the concept of total gastronomy or techno-emotional cuisine
Birth of the concept of sensory anarchy and vindication of insipidity to explain new recipes not always understandable (or desired) by the diner

## First Paradigm: Popularizing the Smell and Taste of Foods

Gastronomy as a social phenomenon emerged during the first half of the nineteenth century after the French Revolution. The most important changes that allowed the conceptual emergence of French cuisine were (10–13): (1) the abolition of the monarchy, which led to the democratization of many of the customs and uses of the Old Regime; (2) the elimination of trade restrictions of the ingredients available for cooking; (3) the development of a powerful guild of experienced but unemployed chefs from the kitchens of the great noble families affected by the revolution, who opened numerous restaurants for an emerging middle class made up of merchants, politicians, journalists, writers, and artists who turned the restaurant into a cultural and social gathering place; (4) the moral transformation of society favoring the arrival at the restaurant of a growing number of hedonistic diners eager to spend money enjoying food; and (5) the social appearance of the gourmet, that in contrast with the traditional gourmand, analyzes, reflects, and philosophizes about food as a benchmark for good living.

The concurrence in Paris between 1800 and 1825 of the great journalist Grimod de la Reyniere, Brillat-Savarin (first gourmet in history), and *Antonine* Carême, the most influential and contradictory chef of the time, represented the birth of contemporary gastronomy (10, 11, 13).

The relevance of the senses linked to chemoreceptors in the cuisine initiated by Carême was reflected in the title of his great epigone Brillat-Savarin's exceptional work “The Physiology of Taste or Meditations of Transcendental Gastronomy” published in 1826, where all his gastronomic experience is collected (12). Perhaps, due to the intuitive nature of smell and taste, and in line with Brillat‘s book, both senses dominated gastronomy for the first half of the nineteenth century.

Smell is the most primitive, but also the most sensitive of all the senses that participate in the gastronomic event. Odorous substances are volatile chemical compounds, usually <300 kDa in molecular weight that activate the olfactory neurons and induce the perception of their smell (14). In the pituitary there are 50 million specific chemoreceptors that are 1,000 times more sensitive than the taste buds of the tongue, so humans have 413 different olfactory receptors that, due to discrimination between their combinations, are capable of perceiving not only the 10,000 classic smells, but up to 1 trillion olfactory stimuli (15).

In addition, smell has a complex post-perceptual processing linked to the olfactory memory, the most extensive after visual memory in humans (14). Due to the connection of smell with the primitive paleoencephalon, a structure already present in fish and reptiles, and its relationship with the olfactory bulb and the diencephalon of mammals (16), the aroma of food is key to memories linked to human nutrition. In this sense, four clarifications must be made:

1. The discovery by man of the varied and powerful range of aromas linked to the release of volatile aromatic compounds by the Maillard reaction, perhaps the most important step in the cognitive evolution of the genus Homo toward the species Homo sapiens (17). This reductive reaction appears when heating a mixture of proteins and carbohydrates (present in the broad sense in any type of meat) at temperatures above 140°C (18) and occurs when heat is used to toast food.
2. Smell is also very fragile and complex because it has a rapid and relatively permanent saturation threshold (19), so the stimulus needs to continuously increase to cause the same excitation. This can be a great physiological obstacle, particularly in the long menus of contemporaneous cuisine, more so when pairing food with numerous wines (5, 20).
3. Smell is the most-likely determinant of feeding behavior in mammals (21). In fact, popular knowledge has always maintained that the wish for foods comes first through smell. Even the appetite and secretion of gastric juices is an immediate result of olfactory stimulation, which was already referred to by Pavlov as the “psychic” phase of digestion, a complex neurophysiological process which has been very recently reviewed (21).
4. The continuous exposure of the pituitary to physical, chemical, or infectious aggressions that occur throughout life and the progressive loss of olfactory neurons in adults results in a gradual loss of smell, even anosmia, which is one of the most common problems of human aging (22). Perhaps for this reason, food enjoyment in the elderly is based in memories of smells (23) and traditional cuisine “foodies” do not crave new gastronomic experiences; it is enough to evoke the powerful aromas and flavors of the past. In fact, people learn to love or to hate certain foods or objects only by appreciating their smell, which is important for the modulation of human behavior, interpersonal relationships and their affiliation to certain groups or social classes (24). And this idealization of the aromas of memory probably is the origin of the hackneyed phrase, very common among food lovers: the best cook in the world is my mother!

Unlike smell, taste is simpler and aversive; in fact, it allows living beings to assure the ingestion of nutrients and reject potential toxics (25). For this reason, almost all mammals, perhaps with the exception of felines (26) respond to the sweet taste since sucrose is an essential source of energy and survival depends on its intake. The perception of sweet, salty, sour, bitter, and umami taste of food is less complex than for odors, although it is more mediated by evolutionary genetic factors and cultural reasons (27, 28): for example, Asian cuisine thrives on umami taste, in Central European and North American cuisine sweet flavors prevail, and in Spain and Italy there is authentic passion for bitter flavors.

Although Carême was mainly acknowledged for the decorative grandeur of his dishes (Pièces Montées) using dough, sugar, jellies, and waxes (13, 29), imitating the great Paris monuments, he was the creator of modern French cuisine (10, 13, 29–31) through:

1. The revision and systematic testing of multiple ingredient combinations, permutations, and mixtures to create multitude of new recipes.
2. The first research on pot au feu (meat stew with vegetables) and related broths which were transferred from popular cooking to academic haute cuisine.
3. The creation of the first experimental kitchen laboratory in history; Carême proposed the chemical analysis of meat broths to understand the role that aromas and taste play in gastronomic pleasure.
4. The edition of the first cooking manuals for the people.
5. Creation of the training, methodological, and documentary foundations of the professional cook.

In summary, Carême was responsible for the transition from the private kitchen of the powerful before the Revolution to the modern public Parisian restoration (13), from culinary art to science and from the cooks' meritorious learning to a professional restaurateur career (30). Carême, as the genial predecessor of Escoffier, embodied this gastronomic paradigm in his colossal *L'art de la cuisine au XIXe siècle* (1833), which covers most of the cooking standards that reached the glory thanks to Escoffier (32) during the second gastronomic paradigm.

## Second Paradigm: Cuisine Rules and the Flavory Sauces of Auguste Escoffier

Although Montagné (first main editor of the *Larousse Gastronomique*) was who initially proposed to eliminate Carême's *Pièces Montées* and their superfluous garrisons, the idea was finally carried out by Escoffier from his golden exile at the Savoy and Carlton Hotels in London, becoming the king of classical French gastronomy (10, 11, 13, 32). Escoffier's influence was so great that many of his proposals (especially those relating to the organization of the kitchen and service at the table) are still valid today.

The main contributions that shaped Escoffier's gastronomic paradigm were successively (10, 11, 13, 31, 32):

1. Simplification of kitchen work with the creation of the *brigades* and *parties de cuisine*.
2. Realization of the first restaurant menu with almost exclusive use of luxury ingredients, like seafood, caviar, truffle, foie gras, poultry, fish, and game.
3. Reduction of the number of menu dishes to an average of 4 with use of a culinary rhetoric based on naming the dishes as a tribute to people: Suprêmes de volaille Jeanette, Sole Dugleré, Tournedos Rossini or Peach Melba.
4. The large number of options in the menu, emphasizing the amplitude and inclusion of seasonal foods, with substitution of the French service (all the dishes served simultaneously on the table) for the Russian service (dishes are served consecutively, following a preset order in the menu).
5. The enormous rigidity of Escoffier's recipes (consommés, potages, creams, *veloutés, brioches, bisques, quenelles*, etc.), which was responsible for the monotonous uniformity of his disciples' cuisine.
6. Finalization of dishes at the table, such as the still current steak tartare with the spectacle in the table service around the sauce boat with the waiter carving, adding sauce and garnishing foods with the ultimate purpose of highlighting the unitary perception of the aromas and tastes of each recipe. And it was precisely the widespread use of complex sauces covering the principal ingredients in numerous dishes from Escoffier which made taste the sensation that best represents the second gastronomic paradigm.

Although the world of sauces was extensively explored by Carême, its systematic study together with the definition of the five essential sauces of classical French cuisine (béchamel, espàgnole, velouté, hollandaise including mayonnaise and tomatoes) is due to Escoffier (33). The main sensory characteristic of all the sauces is their very different flavor, a set of sensations which according to International Organization for Standardization (ISO) can be defined as a “complex combination of the olfactory, gustatory, and trigeminal sensations perceived during tasting. The flavor may be influenced by tactile, thermal, painful, and/or kinesthetic effects” (34). In this context, it should be noted that the texture (essentially the viscosity) of Escoffier's sauces was essential to their perfection. And although classical physiology has always considered that aroma, taste, and kinesthetic perceptions were much more important than texture, sight, and sound in the neurophysiological interpretation of flavor (35), several scientific contributions, including those made by Spence, have emphasized the very notable role that texture, sight and sound play in the evaluation of food flavor (7, 36–41).

The universalization of the proposal of Escoffier (who left more than 20,000 disciples scattered all over the world) occurred with the hatching of the Michelin red guide, especially since 1933 when it included a list of restaurants classified by their famous stars, among which chefs were basically the most famous disciples of the master (42). Thus, the chef became a paid craftsman who reproduced Escoffier's recipes, while the owner of the restaurant acted as maître-restaurateur (13), organizing the establishment, frequently finalizing the dishes at the table (steak tartare, covering meat or fish in sauce, etc.).

In summary, it can be said that Escoffier's contributions to gastronomy were revolutionary in form, but conservative in content, perhaps due to Victorian puritanism (13). However, thanks to his ability to specify Carême's proposals and his synthetic vision of modern gastronomy, Escoffier was fundamentally remembered for having completely transmuted Nature into Culture (43) by cooking raw foods using heat and adding sauces as an essential complement to each dish.

## Third Paradigm: the French Nouvelle Cuisine and the Revolutionary Visual Presentation of Foods

After Escoffier's death in 1935, the French haute cuisine languished for decades resting on the network of schools and professional associations of chefs created from his proposals. As defined by Beaugé (9) “Until Nouvelle Cuisine, the overall impression was that, except for a few exceptional chefs with outstanding skills, new recipes were responses to public demands.” It was the anti-authoritarian protests of May 1968 in Paris and the appealing to the four slogans of truth, lightness, simplicity, and imagination that characterized this movement who facilitated the radical emergence of Nouvelle Cuisine (13).

The fundamental reason for the emergence of Nouvelle Cuisine leaders such as Guèrard, Senderens, the Troisgros brothers, Chapel and Bocusse (all of them great chefs integrated into the system) was their refusal to continue being kitchen artisans as proposed by Escoffier (9, 13, 44), to become the new philosophers of contemporary gastronomy, capable of creating innovative dishes (44) by combining the position of chef with that of entrepreneur (13). And to harmonize and spread this revolution, Nouvelle Cuisine also found its own reporters: Henri Gault, Christian Millau, and his mythical gastronomic guide (45).

The key to the culinary paradigm developed by these chefs and critics was to put personal culinary philosophy and the sensory evaluation of food before the rigid rules designed by Escoffier. “To achieve culinary glory, it is necessary to unlearn what is taught by the classic cooking schools” argued its proponents, and for this Gault and Millau designed the 10 Commandments of Nouvelle Cuisine (13):

1. Thou shall not overcook. This applies to almost all the products used (and abused) by classical cuisine: fish, shells, seafood, game, waterfowls, poultry, which were overcooked (overcooking protects from poisoning due to long and poor storage conditions).
2. Thou shall use fresh, quality products. Select products only if you are sure of their outstanding quality, avoid intensive agriculture.
3. Thou shall lighten thy menu to emphasize and preserve the freshness of the food as much as possible.
4. Thou shall not be systematically modernistic. Avoid a new orthodoxy.
5. Thou shall seek out what new technology can bring to you. This will also improve the cooks' working conditions, through airing and ventilation, and reducing consumption of coal or wood, which are replaced by electrical or gas stoves.
6. Thou shall abolish white and brown sauces, which are heavy and indigestible. Also, abolish marinated dishes and high game.
7. Thou shall not ignore dietetics. The postwar times of malnutrition are over.
8. Thou shall not cheat on thy presentation. Simplicity instead of fakery.
9. Thou shall be inventive.
10. Thou shall not be prejudiced.

Some of the immediate consequences of implementing the 10 commandments of the Nouvelle Cuisine were:

1. Substitution of terminology employed by Escoffier, with novel names for dishes that are impregnated with feeling, evocation, and poetry, based on the most original presentation possible of the ingredients in the dish (13). For the Nouvelle Cuisine the visual impact of the dishes was as important or more than their aroma and taste because it is well-known that in foods the implicit visual capture is fundamental to make them appetizing and the visual capture is only modulated by experience (46): food must first enter via the eyes. In fact, the first visual impression of food is to a large extent decisive for the perception of its flavor (47).
2. In connection with the previous point, the Nouvelle Cuisine was first to propose the trompe l'oeil (an illusion to deceive the senses) as an effective way of surprising the diner eager of new experiences. And so it proposed to continually transgress the rules of classical cooking (13) through the use of innovative techniques for fish and seafood cuts (i.e., carpaccio), instead of traditional transversal slices, or proposing dishes with antagonist ingredients, like land and sea (meat and fish), foie gras, and vegetable salad or the use of sweet foods for starters and salty foods for desserts, etc.
3. According with the sixth commandment, sauces were lightened and flour, butter, cream, and eggs were eliminated to help their digestion; the use of thick sauces comes to an end with a reduction of ingredients and cooking time; and raw foods are introduced in search of maximum naturalness and preservation of their nutritional qualities (48).
4. Increase in the number of dishes in the menu introducing an extensive sampling of minimal portions for each dish, and according to the seventh command, seeking to limit caloric intake (cuisine minceur) thus making the long and narrow menu compatible with a sedentary lifestyle (48, 49).
5. To develop the ninth commandment, the new chefs introduce several ingredients not used before in haute cuisine, such as wild aromatic herbs, new spices, tropical fruits, and very low-priced fresh fish and vegetables (13), like Carême did in the nineteenth century by bringing the pot au feu of the people to the tables of the bourgeoisie and nobility. In addition, traditional Mediterranean and eastern recipes (risotto, tajin, sashimi, etc.) are incorporated into haute cuisine, as well as the introduction of fusion cuisine for the first time in history (50).
6. The chef becomes the restaurant's lead figure to the detriment of the maître and waiters, whose protagonism is diminished (13). Thus, recipes are no longer completed at the table, and the dishes are presented fully finished (Kaiseki Japanese style service), in a specifically decorated dish and even covered by a bell, seeking to visually surprise the diner similarly to what Japanese ceramics propose (51).

In summary, the Nouvelle Cuisine means the emergence of a new intellectual and terminological discourse, reflected in the name of the dishes (“floating truffle garden Island with hot puree over cold pea soup” by Michel Guèrard), and where the visual presentation of food is increasingly risky, being impressionist painting the art that could best represent this movement.

## Fourth Paradigm: Culinary Abstraction and the Textural Revolution in Gastronomy

Abstract art does not attempt to represent external reality, but rather to achieve its effect using shapes, colors, and textures. And just as abstraction in painting displaced impressionism and expressionism, the abstract gastronomic revolution realized in Spain by Ferrán Adrià displaced the preeminence of French Nouvelle Cuisine from the map. It was Adrià himself, a perfect example of an entrepreneur with exceptional innovative capacity (52), whom from his restaurant El Bulli in Roses (Girona) established the conceptual field for abstract cuisine: “Cooking is a language through which all the following properties may be expressed: harmony, creativity, happiness, beauty, poetry, complexity, magic, humor, provocation, and culture” (53).

And it is in this context that, in addition to vision, the manipulation of food to stimulate touch and hearing with new sensations begins to play an increasing role in gastronomy, with a relative displacement of smell and taste as the main senses in the evaluation of flavor (36–38). In this way can be understood the growing complexity of the gastronomic act, which moves toward the multifactorial perception of experience and has become a fundamental component of twenty first century gastronomy (5–7, 37). The culinary abstraction began in 1994 from Adrià's version of the classic Spanish vegetable stew (textured vegetable panache) and occurred in three consecutive phases: (1) deconstruction, (2) constructionism, and (3) new experimental cuisine.

### Deconstruction in Cooking

In philosophy, the term deconstruct (Jacques Derrida) appears as an essential objection to the hierarchical binary system of antagonism that characterizes Western society, where language and writing, mind and body, religion and secularism, literality and metaphor are counterposed (54). In this context, Derrida's deconstruction broke boundaries, blurred concepts, and provided different views on the same issue. This way of analyzing problems has classically been called postmodernism (55).

To understand Adrià's purpose when deconstructing food, his own reflections referred by Parasecoli (56) are insightful: “Deconstruction in the kitchen consists of using (and respecting) already known harmonies, transforming the texture of ingredients, as well as…the intensity of its flavor.” In fact, Adrià used the term deconstruction not only as an alternative to impress the diner (philosophy inherited from the Nouvelle Cuisine), but more importantly, “undoing [food] analytically to favor its sensory perception,” physically changing food's texture rather than modifying its chemical nature (its perceived aroma and taste). And so are born the new ice creams, mousses, gelatins and foams, first cold and then hot (first use of synthetic emulsifiers such as lecithin in haute cuisine!), which characterized the dishes of the early El Bulli era (52, 56).

Whereas, in philosophy almost everything is primarily rational with predominance of visual or auditory impressions, in gastronomy the opposite applies: first it is perceived through smell, taste, and the tactile and temperature mouth receptors; then it is intellectualized (57). Adrià's deconstruction can be summarized paraphrasing Lampedusa in *The Leopard*: “if we want everything to stay the same, everything must change” (58), but adding a prodigious range of new textures. This fact which distinguishes Adrià from Picasso, who went much further in his concept of cubism and who said: “for me, each of my abstract paintings is a sum of destructions” (59) and, conceptually, brings this great chef Adrià closer to artists like Dalí (60).

### Molecular Gastronomy and Food Texture

The idea of changing food texture to impress the diner is central to the new science that contributed to the universalization of abstract cuisine. In support of the abstraction in cuisine appears the so-called molecular gastronomy (3, 61–75), created in the 1980s by the British physicist Nicholas Kurti, Physics Professor at Oxford University (61) and Hervé This (63, 64). Initially, molecular gastronomy included modeling recipes, testing old wives' tales, inventing new dishes and introducing new tools, methods and ingredients in the kitchen (63). It was recognized that all recipes are made of two parts that should be studied in their own right. First, the definition of the dish. The second part comprises detailed descriptions of the processes involved in preparing the dish, technical indications along with old wives' tales, proverbs, sayings, and so on. This called these “culinary precisions,” or, for short, “precisions” (64).

Molecular gastronomy subsequently was redefined by This himself (65) and Barham (70) as “a branch of food science, especially but not exclusively concerned with the transformation of foods after cooking and their subsequent consumption. It is applied to culinary rather than industrial processes. In other words, molecular gastronomy is the application of scientific principles to the understanding and improvement of domestic and gastronomic food preparation.” In summary, molecular gastronomy could be the result of transferring physical and chemical knowledge from the laboratory to the kitchen, modifying the sensory perception of food (67, 71–75).

For Adrià himself the philosophy of manipulating or deconstructing food using industrial utensils deserved the denomination of conceptual cuisine, proposing its relationship with the art of the same name (76). In conceptual art, the thought that the artist wishes to express is actually more important than its material representation (77), whereas in Adria's cuisine the food always maintained a positive connection with the visual, sapid, and olfactory memory of the diner (remember the use in El Bulli of bouquets of aromatic herbs served together with the dish to stimulate the sense of smell). The disconnection between cooking, sensations and memory only occurred in the twenty first century with the emergence of purely experimental cooking (see below). Yet in the cook's molecular gastronomy proposal the changes in the aroma, taste, and texture of foods pretended to elicit emotion through the reverberation that gastronomic pleasure produces in the brain via nerve impulses from the sensory organs (78). For this reason, molecular gastronomy was still indispensable in the culinary preparations of any great contemporary chef at the end of the twentieth century. Some authors argued that this type of practice should be called molecular cuisine rather than molecular gastronomy, since the latter would be an exclusively scientific discipline (67, 71).

An example of how molecular gastronomy approaches problems inherent to food technology is exemplified by the relationship between foie gras quality, the histological type of steatosis induced in duck liver, the presence of fat percolation phenomena and animal feed used (79). Most molecular gastronomers are basic scientists, although paradoxically the bible of molecular gastronomy was written by Harold McGee, author and journalist (80).

Perhaps the first direct and concrete application of scientific knowledge to culinary recipes was the note by note gastronomic proposal developed by This and Gagnaire (a pioneer in molecular cuisine together with This) at the end of the twentieth century. The note by note protocol basically consists in the use of synthetic substances and extracts to obtain the smell, taste, and texture of dishes (81), although more with a view to maintaining the sustainability of future molecular haute cuisine in view of a growing lack of ingredients, than for pure conceptual reasons.

In 2004, Nature magazine summarized in an excellent commentary something that many scientists had already proposed: most foods are complex, heterogeneous molecular systems whose behavior obeys the laws of physics, more so than many other materials (82). For this reason, the essential food quality in the fourth gastronomic paradigm is texture, a collective property of complex soft foods, influenced by their temperature, viscosity, and surface tension as well as their structure, composition, and density (83, 84). For these reasons molecular gastronomy produces an unlimited variety of new textures using different techniques and instruments (Thermomix, siphons, Rotaval, vacuum cooking-Gastrovac, Roner incubator, Paco-jet, encapsulators, lyophilizers, viscometers, chromatographs, liquid nitrogen, etc.) Most of these formulas are unstable, with only a few minutes' life, which contributes to their uniqueness and difficult reproducibility (2, 3, 75, 85).

The texture of food determines its mastication and controls the sequence and intensity of flavor arrival to their specific receptors and of aromas to the pituitary via the retronasal pathway, with simultaneous participation of other sensorial modalities (78), tasting flavor and experiencing texture and sound as well, such as hearing and feeling crunchy-crispy o hearing-touch foods (86, 87) to which may even contribute the listening of pleasing music during the meal (88–90).

### Culinary Constructionism

The concept of culinary constructivism was first proposed by This in the late 1990s to endorse its suggestion to add new ingredients and extracts to gastronomy, and was likewise broadly redefined in 2009 (91). It was Adrià however the first to put into practice a concept of “culinary constructivism” at the restaurant to imitate constructivism in art (92). In this way, around 1999 Adrià began experimenting with the use of numerous texturizing polymeric formulas (agar-agar, alginates, xanthan gum, and gellan, etc.) that only reveal the essence of things. Thus, Adrià began to change food textures, building new polymeric vehicles for some of the most traditional ones (agar-agar spaghetti, melon caviar, spherical olives, etc.), all to delight the diner by combining primitive memory images and flavors with surprising new polymeric textures (75, 85). After this creative extravaganza at El Bulli, in August 2003, the New York Times (93) proclaimed Ferrán Adrià the best and most influential chef in the world, perhaps the most important after Escoffier. And so arose an almost infinite range of new food textures based on hot agar-agar gelatin, spherification with alginates, whipped foams with pectins (airs), sticking proteins with transglutaminase, texts, inks, and edible photographs, etc. (2).

In this context and due to its unparalleled naturality, freezing food in liquid nitrogen has been a spectacular culinary construction proposal. Although, the use of liquid nitrogen in the preparation of ice cream in front of students is a classic in education (94), it was not until the end of the twentieth century that This and Gagnaire in their demonstrations of molecular cooking, as well as Heston Blumenthal at The Fat Duck restaurant (Bray, Berkshire, UK) introduced its use in haute cuisine for the preparation of desserts and ice creams (75). However, the systematic use of cooling in liquid nitrogen to radically change the texture of salty foods was the work of young chef Dani García at the Tragabuches restaurant in Ronda, Spain, in 2004 (95), followed immediately afterwards by Ferrán Adrià at El Bulli (85). By ultra-freezing in liquid nitrogen, García and Adrià took the idea of surprising the diner without altering the olfactory and gustatory properties of the food to the extreme (85).

The evolution of constructivism ended up applying the use of polymers to imitate within the dish the geographical environment that inspired the chef to create it, whose best ideologists besides Adrià have been chef Quique Dacosta in his restaurant in Denia (Spain); Gualtiero Marchesi in Erbusco Italy), and Massimo Bottura in La Francescana, Modena (Italy) with multitude of imitators. The so-called edible landscapes which supposed the reincorporation of the decorative spirit of Carême's Pièces Montées, now completely eatable, inspired by the comestible landscape photographs previously devised by Carl Warner (96), an emerging form of the so-called new tourism (97).

### The Experimental Cuisine and Sustainability Cooking of The Twenty First Century

Ferrán Adrià and other culinary abstraction chefs made most of their dishes by intuition and personal genius, using an industrial technology which was largely unknown to them. However, the techno-cuisine, as it was currently interpreted, has gone further by proposing (67): (1) an extensive use of both scientific knowledge of food preparation and technological devices which were originally developed in the context of low-cost, industrial food manufacturing and (2) the systematic use of chemical reactions and physicochemical phenomena in foods to develop new recipes which cannot be prepared using standard kitchen utensils.

With a merely expository intention the experimental cuisine could consecutively be subclassified in (a) Techno-cuisine including neurogastronomy, gastrophysic, and gastronomic engineering and (b) Conceptual and intellectually designed cuisine.

- Techno-cuisine
  An inevitable consequence of the progressive technification in the kitchen is its progressive geographical decontextualization in the sense evoked by Winet (98), where the techno-cuisine would be a static universal practice devoid of any kind of local influence, in a similar way to what happened with the French academic cuisine of Escoffier, which spread throughout the world for more than 80 years as the only possible option in the most famous restaurants of the time (13). In the twenty first century and due to various influences of fusion cuisine and the sustainability of haute cuisine, the decontextualization proposed by techno cuisine has quickly been replaced by various movements that advocate the local rooting of haute cuisine in order to make it more sustainable (see below).
  The techno-cuisine, as it was proposed by Heston Blumenthal at The Fat Duck at the end of the twentieth century (99) went so far as to propose the creation of a controversial figure, the technochef, which presupposed the cook's apprenticeship before starting out as a creator of new recipes for many of the culinary concepts analyzed by gastronomic science at the end of the twentieth century. In this process it would be necessary for technochefs to acquire knowledge of neurogastronomy (36, 100), gastrophysics (101), gastronomic engineering (2, 102) as well as basic nutritional knowledge, digestive physiology, and psychology (99).
  The techno-cuisine promoted for the first time the use of chemical molecules in dishes to directly stimulate smell and taste, while sounds played via headphones, stimulating the diner while eating (99, 102), which has a solid experimental basis (103). This was the beginning of the so-called techno-emotional cuisine, term coined by gastronomic journalist Pau Arenós (2, 75, 104, 105) or experimental cuisine (101), term adopted in this revision in agreement with Spence, which aimed to achieve “total gastronomy,” understood as the ordered, simultaneous, and conceptualized stimulation of all the senses and brain circuits possible during the gastronomic act, which incorporated as many artifices as necessary (magic games, play-food, *ad hoc* musical compositions, etc.). In this techno-emotional cuisine trompe l'oeil was the rule; the technical purity, the norm. It is interesting that Adrià himself, one of the creators of techno-emotional cuisine, soon reneged on it in order to claim the role of the chef as a simple cook, if not more an artist and technologist who created the El Bulli brand, than a scientist (85).
- b) Conceptual and Intellectually designed cuisine
  In the first half of the twentieth century, abstract expressionism dominated pictorial art (106). In response to the idyllic contemplation proposed by the abstract expressionist, at the end of the sixties arose conceptual art (107), which elicited only the most enthusiastic acceptance or the deepest rejection. For the conceptual artist the key is the ideas, putting the narrative before their formal, aesthetic, and technical aspects (108). Thus, body art and tattoo, videographic expression, theatrical representations or performances, and artistic installations arose consecutively. Like the artistic performances in conceptual cuisine the important thing is to tell stories with food, to recreate sensory experiences, rather than the gastronomic or artistic fact itself (109, 110).
  Initially, the theatricalisation of stories around the culinary act, which was already suggested at the height of experimental cooking (111), led some pioneering chefs at the beginning of the twenty first century to prepare dishes containing sensory installations whose ultimate purpose was to be eaten (this is the perfect connection with the ephemeral nature of art performances), and whose aim was to transfer the sensory experience from the chef to the diner in a didactic and direct way. In culinary sensory installations, unlike abstract cooking, the concept takes precedence over technique (112), allowing the dynamic production of dishes in front of the diner, reminiscent of Escoffier's cuisine, as is the case with many of the dishes made with liquid nitrogen (85), and creating a spectacle through playful gastronomy, in which in 1996 Adrià's Spices dish in El Bulli was also pioneering (112). Other examples of sensory installations have been proposed by the Roca brothers at the Celler de Can Roca (Girona, Spain), such as their memorable mussels with Riesling which aim to show the influence of wine in their cuisine; the fun edible apple balloon by Achatz in Alinea (Chicago, USA) or the baroque vegetable marrow salad with seafood, cream of farmhouse lettuce and iodized juice by Martín Berasategui (Lasarte, Spain), perhaps the chef who best combines numerous ingredients within the same dish with unsurpassable aesthetic and flavor harmony.
  In classical gastronomy, and even in contemporary gastronomy led by Adrià, it was assumed that in gastronomy one first feels through sight, smell, taste, tactile, and somatosthetic oral receptors, and even hearing, and then one intellectualizes the experience (3, 7, 7, 20, 37, 55, 72, 75, 91). The idea that in gastronomy the coordinated perception of all sensations (5–8) was the key to techno-emotional cuisine was always assumed by chefs and foodies at the end of the twentieth century. However, Pierre Gagnaire, a pioneer in molecular cuisine together with Hervé This, can also be considered a champion of a new proposal of intellectually designed cuisine currently fashionable in some restaurants. In fact, Gagnaire was compared to Kandinsky when in 2006 he took another step in culinary abstraction by dispossessing food of all its sensory qualities except flavor by the use of dyes such as carotenoids or chlorophyll (113), to obtain fish that looks like a vegetable or orange Italian pasta, or through alternative carving techniques, which transform the shapes of traditional ingredients (square fish!), all with the ultimate goal of surprising and exciting the diner.
  This line of deceiving (rather than surprising with trompe l'oeil) the senses in order to move the diner when the experience is orally related to him is but another twist to the concepts of deconstruction, constructivism and techno-cuisine and has been extensively analyzed in the psychology of the senses, especially with regard to the color of the food ingested (114, 115). After his revolutionary proposal, Gagnaire has always summarized his cuisine with the idea that the ingredients and the images of a dish do not always provide a recipe, they only give a hint (116). Thus, Gagnaire's cuisine was transformed into a conceptual exhibition with elaborate explanatory narrative that, being devoid of recognizable objects, was only subject to the emotional judgment of its author, which would elicit only enthusiastic adhesion or rejection by the client. In this context, although with an ecological background linked to the surrounding nature, the cuisine of Andoni Luis Adúriz could be framed in the Mugaritz restaurant in Rentería (Spain), which, after paradoxically proclaiming the insipidity (no-taste of foods) as a property worthy of praise for his dishes, has ended up introducing “gastronomically damned flavors” (“the moldy apple”) as one of the basic characteristics of his cuisine (117). At that time, the Mugaritz diner was faced with the dilemma of submission or rebellion (supposedly to his proposals) when the diner sat at the table to the tasting menu proposed by the chef (of course, in this type of restaurants there is no à la carte menu!).
  Perhaps the greatest obstacle that this type of cuisine has encountered in the general public is that on numerous occasions the chef pretends that, in addition to appreciating his genius, the diner incorporates their new proposals into his taste memory. These chefs seem to forget that the olfactory, taste, and visual memory of each diner is essential to recognize or induce pleasant or unpleasant stimuli in the proposed experience (36, 118–120) which makes it difficult for the diner to evaluate the experience as positive if there is negative antecedents in its memory. Faced by these experimental findings some of the most radical chefs of the twenty first century refuse to taste the recipes where they exhibit their art.
  To avoid eventual negative diner responses or simply the unwillingness to try new experiences, it is possible to condition the perception of the diner by restricting some of his sensations, especially his sight, with food tasting in complete darkness, which can open other perspectives on the perception and effect of what has been ingested (121, 122).
  In summary, the fourth paradigm consecutively contributed: (1) Food deconstruction, which aims to change its texture without diminishing its aroma and flavor; (2) The creation of new food textures, that becomes one of the sensory keys of gastronomic pleasure; (3) The introduction in haute cuisine of new texturizing and emulsifying molecules of industrial origin as ingredients; (4) Involvement of the senses of sight (edible landscaping) and hearing in gastronomic pleasure; (5) Food reconstruction that pursues a radical modification of its flavor (melon caviar) or its texture (spherical olives, agar-agar spaghetti); (6) The introduction of liquid nitrogen to create new textures and temperatures; (7) Development of the concept of total gastronomy or techno-emotional cuisine; and (8) Birth of the concept of intellectually designed cuisine and vindication of insipidity to explain new recipes not always understandable (or desired) by the diner.

## The Global Socialization of Gastronomy: Toward a Fifth Paradigm?

After the emergence of social networks and globalization, the internationalization of the most remote culinary uses and the great economic crisis of 2008, a radical change has begun in the culinary tastes of Western society (123). Among the new gastronomic practices are:

1. Low cost restaurants, adapted to the decreasing purchasing power of the western middle class, which is however increasingly open to new gastronomic experiences, on a tighter budget (124).
2. The conceptual internationalization of the Spanish tapa (125) as an informal gastronomic practice, becoming the foundation of gastrobars or gastro pubs, many of them created as second brand by the great chefs of the moment, and where the public enjoys the media myth at a discount.
3. The growing role of the mass media in developing trends, where gastronomy is no exception, and where it is crucial that gastronomic opinion is based on the experience of the best communicators and cooks of the time (as happened in the 60s in the USA with the Julia Child's program, The “French Chef” or currently with the “Ultimate Cookery” Course). However, in the only scientific study published on the subject, any positive effect was more on the finances of the media restaurants themselves than on the gastronomic quality of the environment (126). Another illustration of the negative influences of mass media is the proliferation of TV shows (Master Chef, The Great British Bake, and so on), where amateurs without the necessary experience are crowned as celebrity chefs, simply because they communicate well and connect adequately with society.
4. The progressive disappearance of the professional gastronomic critic (not exempt from elitism and in many cases in close relationship with the most famous chefs) and its replacement by a plethora of social media influencers (127, 128), who interact in real time on the Web, and who are frequently biased by hidden commercial interests.
5. The meteoric growth of ethnic cuisine that, in addition to providing new gastronomic experiences at low cost (129), allows some luxury restaurants of the Michelin guide to innovate through creative fusion (130), especially with Chinese, Japanese, Mexican, and Peruvian cuisines. In this sense the non-reflective mix of world's food can end in con-fusion more that an authentic fusion in the sense advocated by Spence (130). Although with a considerable risk of geographical decontextualization (98), the development of this type of cuisine is fully in line with the increasingly cosmopolitan spirit of twenty first century society.
6. The growth of local ecological cuisine and the consequent locavorism, which aims to use locally-grown ingredients (131), in direct opposition to the universal proposal of the abstract cuisine of the late twentieth century and where some restaurants like Noma in Denmark have come to be recognized as a world reference. Moreover, a very positive aspect of locavorism is that it fosters sustainable gastronomy strengthening food resources in the area, supporting local employment, and eliminating environmental costs associated to food transportation (132–134).
7. The development of numerous formulas of dietary neo-cuisine (devoid of allergens, vegan, crude, paleo-cuisine, etc.) (123), very limited in ingredients and culinary technology, and the booming fashions of comfort food (135) or smart food (136) as gastronomic formulas.
8. The gastronomic egocentrism associated with social networks and the self-itis linked to photography (137) or foodstagramming (138), which tends to give more value to the image of the photographed dishes than to their gastronomic importance.

Will it be possible that the coexistence between antagonistic tendencies such as the creative haute cuisine that still survives and the new gastronomic practices be what characterizes the emerging fifth paradigm? Perhaps the socialization of gastronomy will end up winning the game against the classic gastronomy based in the Michelin Guide. In this sense, the ultramodern The World's 50 Best Restaurants List (139), where informal neo-cuisine restaurants, including street food restaurants gain more prominence; and in the growing implementation as a preferred tool for gastronomic consultation of the web portals, which more than the search for gastronomic classic excellence proposes the good, beautiful and cheap triad as its most important gastronomic goal.

## Author Contributions

RM is the sole author of this mini review, which is based on his own experience as a gastronomic critic of the newspaper El Mundo and the Spanish guide The Best of Gastronomy between 1999 and 2011 and as a regular visitor of the best Restaurants of the world until this year.

### Conflict of Interest

The author declares that the research was conducted in the absence of any commercial or financial relationships that could be construed as a potential conflict of interest.
